# Supplementary material for: Drosophila pVALIUM10 TRiP RNAi lines cause undesired silencing of Gateway-based transgenes
Source: Life Sci Alliance. 2022 Nov 29;6(2):e202201801. doi: 10.26508/lsa.202201801 (PMC9711858; doi:10.26508/lsa.202201801)
Supplement: Supplementary file 5 [file LSA-2022-01801_TableS1.docx]

Supplementary Table S1. List of *Drosophila* lines

| ***Short name*** | ***Line Genotype*** | ***Source or reference*** | ***Identifier*** |
| --- | --- | --- | --- |
| ***nub>mRFP*** | w; P{w[nub.PK]=nub-GAL4.K}2, P{w[+mC]=UAS-myr-mRFP}1/CyO | Erkelenz et al., 2021 | originated from RRID:BDSC_63148 |
| ***BuGZ^FlyFos[v318366]^*** | FlyFos016907(pRedFlp-Hgr)(CG1791232384::2xTY1-SGFP-3xFLAG)dFRT | VDRC, Sarov et al., 2016 |  |
| ***nub>mRFP; BuGZ^FlyFos[v318366]^*** | w; P{w[nub.PK]=nub-GAL4.K}2, P{w[+mC]=UAS-myr-mRFP}1/CyO; FlyFos016907(pRedFlp-Hgr)(CG1791232384::2xTY1-SGFP-3xFLAG)dFRT | this study |  |
| ***BuGZ^RNAi[TRiP.JF02830]^*** | y[1] v[1]; P{y[+t7.7] v[+t1.8]=TRiP.JF02830}attP2 | BDSC | RRID:BDSC_27996 |
| ***BuGZ^RNAi[KK104498]^*** | w; UAS-BuGZ^RNAi[KK104498]^ {30B} | VDRC |  |
| ***Lip4^RNAi[TRiP.HM05136]^*** | y[1] v[1]; P{y[+t7.7] v[+t1.8]=TRiP.HM05136}attP2 | BDSC | RRID:BDSC_28925 |
| ***myc^RNAi[TRiP.JF01761]^*** | y[1] v[1]; P{y[+t7.7] v[+t1.8]=TRiP.JF01761}attP2 | BDSC | RRID:BDSC_25783 |
| ***myc^RNAi[TRiP.HMS01538]^*** | y[1] sc[*] v[1] sev[21]; P{y[+t7.7] v[+t1.8]=TRiP.HMS01538}attP2 | BDSC | RRID:BDSC_36123 |
| ***myc^RNAi[GD2948]^*** | w;; UAS-myc^RNAi[GD2948]^ | VDRC |  |
| ***Actβ^RNAi[TRiP.JF03276]^*** | y[1] v[1]; P{y[+t7.7] v[+t1.8]=TRiP.JF03276}attP2 | BDSC | RRID:BDSC_29597 |
| ***ftz-f1^RNAi[TRiP.JF02738]^*** | y[1] v[1]; P{y[+t7.7] v[+t1.8]=TRiP.JF02738}attP2 | BDSC | RRID:BDSC_27659 |
| ***yki^RNAi[TRiP.JF03119]^*** | y[1] v[1]; P{y[+t7.7] v[+t1.8]=TRiP.JF03119}attP2/TM6B | BDSC | RRID:BDSC_31965 |
| ***Atf3^RNAi[TRiP.JF02303]^*** | y[1] v[1]; P{y[+t7.7] v[+t1.8]=TRiP.JF02303}attP2 | BDSC | RRID:BDSC_26741 |
| ***DH44^RNAi[TRiP.JF01822]^*** | y[1] v[1]; P{y[+t7.7] v[+t1.8]=TRiP.JF01822}attP2 | BDSC | RRID:BDSC_25804 |
| ***brat^RNAi[TRiP.HM05078]^*** | y[1] v[1]; P{y[+t7.7] v[+t1.8]=TRiP.HM05078}attP2 | BDSC | RRID:BDSC_28590 |
| ***mago^RNAi[TRiP.HM05142]^*** | y[1] v[1]; P{y[+t7.7] v[+t1.8]=TRiP.HM05142}attP2 | BDSC | RRID:BDSC_28931 |
| ***pUWG-RNase H1*** | w;; pUWG-RNase H1/TM6B | this study |  |
| ***nub>mRFP; pUWG-RNase H1*** | w; P{w[nub.PK]=nub-GAL4.K}2, P{w[+mC]=UAS-myr-mRFP}1/CyO; pUWG-RNase H1/TM6B | this study |  |
| ***pUWG-mCherry*** | w; pUWG-mCherry/CyO, P{ActGFP}JMR1 | this study |  |
| ***pUWG^ΔattB^-mCherry*** | w; pUWG^ΔattB^-mCherry/CyO, P{ActGFP}JMR1 | this study |  |
| ***pUWG-mCherry, nub>*** | w; P{w[nub.PK]=nub-GAL4.K}2, pUWG mCherry/CyO, P{ActGFP}JMR1 | this study |  |
| ***pUWG^ΔattB^-mCherry, nub>*** | w; P{w[nub.PK]=nub-GAL4.K}2, pUWG^DattB^ mCherry/CyO, P{ActGFP}JMR1 | this study |  |
| ***SmD3::3xHA^[FlyORF F003987]^*** | yw;; pGW SmDS3::3xHA attP 86Fb/TM6B | FlyORF F003987 |  |
| ***nub>mRFP; SmD3::3xHA^[FlyORF F003987]^*** | w; P{w[nub.PK]=nub-GAL4.K}2, P{w[+mC]=UAS-myr-mRFP}1/CyO; pGW SmDS3::3xHA attP 86Fb/TM6B | Erkelenz et al., 2021 |  |
| ***w^1118^*** | w[1118] | BDSC | RRID: BDSC_3605 |
