# Supplementary material for: Drosophila pVALIUM10 TRiP RNAi lines cause undesired silencing of Gateway-based transgenes
Source: Life Sci Alliance. 2022 Nov 29;6(2):e202201801. doi: 10.26508/lsa.202201801 (PMC9711858; doi:10.26508/lsa.202201801)
Supplement: Supplementary file 6 [file LSA-2022-01801_TableS2.docx]

Supplementary Table S2. List of oligonucleotides and plasmids

**Primers used for cloning**

| ***Name*** | ***Sequence (5′-3′)*** | ***Purpose*** |
| --- | --- | --- |
| ***mCherry fwd*** | TTACTTGTACAGCTCGTCCATG | Cloning of mCherry into the pENTR/D-TOPO |
| ***mCherry rev*** | CACCATGGTGAGCAAGGGCGAGGA | Cloning of mCherry into the pENTR/D-TOPO |
| ***delAttB Frag1 fwd XhoI*** | CGCACTCGAGCATTTGTGTGC | Deleting the attB sites from pUWG-*mCherry* |
| ***delAttB Frag1 rev*** | AAGGGGGCGGCCGCGGTGATGCTGAATTCCTGCAGC | Deleting the attB sites from pUWG-*mCherry* |
| ***delAttB Frag2 fwd*** | GCAGGAATTCAGCATCACCGCGGCCGCCCCCTTCACC | qPC Deleting the attB sites from pUWG-*mCherry* |
| ***delAttB Frag2 rev*** | TGCTCACCATGCCATCAGCGGCGCGCCCACCCTTTTACTTG | Deleting the attB sites from pUWG-*mCherry* |
| ***delAttB Frag3 fwd*** | AGGGTGGGCGCGCCGCTGATGGCATGGTGAGCAAGG | Deleting the attB sites from pUWG-*mCherry* |
| ***delAttB Frag3 rev XbaI*** | ACGTCTAGACTAGCTTACGTCAATT | Deleting the attB sites from pUWG-*mCherry* |
| ***RNase H1- fwd SalI*** | ACAAACCATGGGAACCAATTCAGTCGACATGTTACTTCCGCGGTATTTTTGCTGG | Cloning of RNase H1 into the pENTR 4 Dual selection |
| ***RNase H1 no TAG rev NotI*** | ACAAAGCGGCCGCGAACCATTTTTCTGCTTATACAAGGCGG | Cloning of RNase H1 into the pENTR 4 Dual selection |

**Plasmids**

| ***Name*** | ***Purpose*** |
| --- | --- |
| **pUWG** | Gateway destination expression vector, poly-ubiquitin promotor, C-terminal GFP, Hsp27 terminator |
| **pUWG-*RNase H1*** | Gateway destination vector expressing C-terminally GFP-tagged RNase H1 |
| **pUWG-*mCherry*** | Gateway destination vector expressing mCherry |
| **pUWG^ΔattB^-*mCherry*** | Gateway destination vector expressing mCherry, attB1 and attB2 sites are missing |
| **pENTR 4 Dual Selection** | Entry vector, Gateway cloning |
| **pENTR/D-TOPO** | Entry vector, Gateway cloning |
